# Inositols in the Treatment of Insulin-Mediated Diseases

**DOI:** 10.1155/2016/3058393

**Published:** 2016-09-08

**Authors:** Giovanna Muscogiuri, Stefano Palomba, Antonio Simone Laganà, Francesco Orio

**Affiliations:** ^1^IOS and Coleman Medicina Futura Medical Center, Centro Direzionale, 80143 Naples, Italy; ^2^Department of Obstetrics and Gynecology, Arcispedale Santa Maria Nuova-IRCCS, 42123 Reggio Emilia, Italy; ^3^Unit of Gynecology and Obstetrics, Department of Human Pathology in Adulthood and Childhood “G. Barresi”, University of Messina, 98125 Messina, Italy; ^4^Endocrinology, Department of Sports Science and Wellness, “Parthenope” University of Naples, 80133 Naples, Italy; ^5^Fertility Techniques SSD, “S. Giovanni di Dio e Ruggi d'Aragona” University Hospital, CMSO (Centro Medico Specialistico Orio), 84131 Salerno, Italy

## Abstract

A growing body of research is currently focused on the role of inositol isomers and in particular myo-inositol (MYO-INS) and D-chiroinositol (DCI) in the treatment of insulin resistance states. Both isomers have been shown to exert insulin-mimetic action and to lower postprandial glucose. Further, insulin resistance-related diseases were associated to derangements in inositol metabolism. Thus, the aim of this review is to provide current evidence on the potential benefits of inositol isomers (MYO-INS and DCI) in the treatment of disease associated to insulin resistance such as polycystic ovary syndrome (PCOS), gestational diabetes, and metabolic syndrome. Finally, molecular insights into inositol insulin-sensitizing effects will be covered focusing on the possible role of inositol glycans as insulin second messengers.

## 1. Introduction

Inositol (Ins), also known as cyclohexane-1,2,3,4,5,6-hexol, belongs to vitamin B complex. Inositol is a polyol that thanks to epimerization exists under nine stereoisomeric forms depending on the spatial orientation of its six hydroxyl groups. Myo-inositol (MYO-INS) and D-chiroinositol (DCI) are stereoisomers of inositol that are currently used for the treatment of polycystic ovary syndrome (PCOS) [1, 2]. 99% of Ins is present in both nature and mammalian cells under MYO-INS form while the remaining 1% is present under DCI form. MYO-INS regulates glucose metabolism and transport [3]. MYO-INS could be found both in free form and as component of the membrane phosphoinositides into the cells [4]. Phosphatidyl-myo-inositol is the precursor of phosphatidyl-Ins phosphate and phosphatidyl-Ins bisphosphate (PIP2) whose hydrolysis results in Ins trisphosphate (Ins-1,4,5P3, InsP3) that plays the role of second messenger taking part in the activities of several hormones such as follicle-stimulating hormone (FSH) and insulin [4, 5]. Moreover, InsP3 binds the membrane receptors of mitochondria and the endoplasmic reticulum, leading to an increase in calcium influx into the cytosol, which activates protein kinase C and mediates cellular responses [4].

MYO-INS plays the role of precursor of phosphoinositides, membrane components, second messenger signalling, and hyperosmotic stress protectant in both germ and somatic cells [6, 7]. The conversion of MYO-INS into DCI is made by NAD/NADH epimerase that is an insulin-dependent enzyme whose activity is the major determinant of the concentrations of these two molecule in hepatic, adipose cells and muscle fibers [8]. The epimerization of MYO-INS is particularly relevant to hepatic and muscle cells, taking part in glycogen synthesis [3].

The role of DCI in insulin signalling pathway is expressed in terms of stimulation of pyruvate dehydrogenase phosphatase, protein phosphatase 2C, and Ins-phosphate glycan [9].

The blunted ratios of increased MYO-INS to decreased DCI in urine have been indicated as marker of insulin resistance in human subjects [8]. In fact, muscle biopsies of diabetic subjects revealed the absence of DCI compared to controls, even after insulin administration and increased levels of MYO-INS, which was further induced by insulin administration [10]. A decrease of 50% of DCI has been found in both urine and hemodialysate of diabetic subjects [11]. It has been hypothesized that this imbalance between MYO-INS and DCI could be due to a defect in MYO-INS to DCI epimerization. In agreement with this hypothesis, a reduction of [^3^H]-MYO-INS to [^3^H]-DCI has been demonstrated in muscle, liver, and fat cytosolic extracts of Goto-Kakizaki diabetic rats compared to Wistar control rats [12]. In the same tissues a 2-fold decrease of epimerase activity has been observed and this was consistent with human evidence [10]. The reduction of DCI results in the decreased availability of inositol phosphoglycan that is a second messenger of insulin and thus contributing to the onset of insulin resistance.

The aim of this paper is to review the current evidence regarding the role of MYO-INS and DCI in the pathogenesis and treatment of diseases characterized by insulin resistance.

## 2. PCOS

PCOS is characterized by reproductive and metabolic implications and it is currently considered as the most common cause of female infertility [13, 14]. In particular PCOS has been associated with several risk factors for cardiovascular disease (CVD) such as obesity, diabetes, hypertension, and dyslipidemia which share the same pathogenetic mechanism, that is, insulin resistance [14–16].

Insulin resistance and compensatory hyperinsulinemia result in an increased ovarian androgen production along with the reduction of hepatic sex hormone binding globulin (SHBG) production. This derangement is responsible for androgen excess [27]. Obesity may contribute to worsening insulin resistance and as a consequence the degree of androgen excess, the reproductive features of PCOS, and the clinical PCOS phenotypes. Insulin resistance has been demonstrated to be an intrinsic feature of PCOS. A clamp study performed in both lean and overweight PCOS women and controls demonstrated that insulin resistance was independent of BMI in PCOS and was present in 85% of PCOS women and, in particular, in 75% of lean PCOS women and 95% of overweight PCOS women [28, 29]. Further, the adverse impact of obesity on insulin resistance was greater in PCOS women than controls. As a matter of fact overweight control women had comparable degree of insulin resistance to lean PCOS women, suggesting that PCOS women are metabolically similar to overweight/obese non-PCOS women [28].

The major defect of insulin action in PCOS patients is probably due to a postbinding defect in insulin receptor-mediated signal transduction that results in a significant derangement in receptor binding [30]. This seems to be an intrinsic genetic defect in PCOS women and consists of increased insulin-independent serine phosphorylation and decreased insulin-dependent tyrosine phosphorylation [30]. Insulin resistance and compensatory hyperinsulinemia might contribute to the spectrum of the disorders related to PCOS by different mechanisms. Insulin may exert its action on pituitary regulating LH secretion, although studies performed on this topic provided conflicting results [30]. Rat pituitary cells preincubated with insulin showed an increased response of LH after GnRH administration while insulin infusion in PCOS women did not change LH secretion or release after GnRH stimulation [30]. Further, the experimental reduction of hyperinsulinemia has been reported to decrease serum LH levels [30], although it is unknown whether decreased serum LH is an effect of decreased insulin levels or of increased ovarian estrogen production due to resumed folliculogenesis. At the peripheral level, insulin resistance acts on hepatic, muscle, and ovarian function. Hyperinsulinemia decreases the hepatic synthesis of SHBG and this mechanism contributes to the increase of the free androgens and consequently the peripheral androgen action; moreover, hyperinsulinemia also blocks the hepatic secretion of the IGF binding protein- (IGFBP-) 1, resulting in an increased bioactivity of IGF-I and IGF-II which are two important regulators of ovarian follicular maturation and steroidogenesis [30]. The ovarian androgen production from theca cells is increased by IGF-I and IGF-II systemic increase that binds IGF-I receptors [30]. The decreased expression of genes involved in mitochondrial oxidative metabolism has been reported at muscular level [31]. Alterations in insulin signaling pathways along with free fatty acid (FFA) metabolism also have been detected at muscular level in insulin resistance state [31]. Hyperinsulinemia prevents follicular development and causes anovulation through two mechanisms: (1) directly acting at ovarian level causing premature follicular atresia and antral follicular arrest [30] and (2) indirectly causing dysfunction of ovarian response to endogenous gonadotropins [30]. In fact women at the time they present complaining of infertility and/or irregular menses already showed adverse metabolic effects of PCO. Obesity has been reported to worsen menstrual irregularity and increase the follicle number and serum total testosterone level [32, 33].

Oral contraceptives (OCs) are considered the first-line treatment for menstrual disturbances and hirsutism/acne in patients with PCOS [34, 35], while the use of oral insulin-sensitizing compounds such as pioglitazone and metformin is suggested for the treatment of hyperinsulinemic condition of PCOS patients [36]. In particular metformin is the most used insulin-sensitizing drug in the treatment of PCOS. Metformin enhances insulin sensitivity inhibiting hepatic glucose production in liver and increasing glucose uptake and utilization in muscle tissue and decreases total and free-testosterone concentrations. However, metformin can induce gastrointestinal side effects, thus decreasing patients' compliance [37]. Further, metformin has been reported to have a beneficial effect in cancer treatment. Patients with early stage breast cancer who were receiving metformin along with neoadjuvant therapy experienced a higher pathologic complete response [38]. Metformin usage was also associated with improved survival of diabetic prostate cancer patients [39]. The possible molecular mechanism for the anticancer effect of metformin depends on the stimulation of AMP-activated protein kinase (AMPK) and its upstream regulator liver kinase B1 (LKB1) that is a well-known tumor suppressor protein [40]. Inositol has also been reported to have an anticancer effect both on prostate and on human colon cancer [41, 42]. The mechanism by which Ins exerts an anticancer effect seems to be related to the ability of inositol to decrease the mRNA and protein expression of PI3K and Akt. Moreover, Ins inhibited the phosphorylation of Akt (pAkt), whereas it increased the expression of its downstream effector, caspase-9, thus suggesting that inositol suppresses cell survival and proliferation by targeting PI3K/Akt pathway [42].

Recently MYO-INS and DCI have been used for the treatment of PCOS. MYO-INS has also been reported to significantly decrease hyperandrogenism (*p* < 0.001) and insulin resistance (*p* < 0.001) in women with PCOS [43, 44]. Further, MYO-INS has been demonstrated to restore spontaneous ovarian activity and thus fertility in most patients with PCOS [45]. A randomized controlled trial was performed in 20 overweight PCOS women that were randomized to receive MYO-INS 2 gr plus folic acid 200 mug or only folic acid 200 mug. Patients taking MYO-INS experienced a significant improvement of reproductive axis (*p* < 0.05) and insulin resistance (*p* < 0.05) state after 12 weeks of supplementation [17]. On such basis, the efficacy of MYO-INS 2 gr for 8 weeks of treatment has been investigated in 42 PCOS obese women. Although all the enrolled subjects improved both hormonal and insulin resistance parameter, PCOS women with fasting insulin levels above 12 *μ*U/mL experienced a greater reduction of both fasting insulin plasma levels and area under the curve of insulin under oral glucose tolerance test compared to patients with fasting insulin levels below 12 *μ*U/mL [18]. A significant weight reduction (*p* < 0.01) along with decrease in leptin levels (*p* < 0.01) has been reported in a double-blinded, placebo-controlled study in which 92 women were randomized to receive 400 mcg folic acid as placebo or MYO-INS plus folic acid (4 g MYO-INS plus 400 mcg folic acid) for 14 weeks of treatment [19]. Artini et al. provided evidence that 12 weeks of treatment with MYO-INS were effective in reducing plasma LH (*p* < 0.005), prolactin (*p* < 0.05), LH/FSH (*p* < 0.01), and insulin resistance measured by HOMA-IR index (*p* < 0.01) [20]. Unfer et al. [2] reviewed and analyzed the six Randomized Controlled Trials (RCTs) that assessed the effectiveness of MYO-INS supplementation in improving PCOS hormonal and metabolic disturbances. A dosage of 2 and 4 g/day was tested for 12 and 16 weeks in those studies and no side effects were reported. They provided level Ia evidence of MYO-INS effectiveness that was mainly based on improving insulin sensitivity of target tissues. This mechanism has a beneficial effect on the reproductive axis, restoring ovulation and improving oocyte quality, and on hyperandrogenism.

DCI has been reported to reduce insulin resistance both in lean and in obese patients with PCOS concurring to improve ovarian function and hyperandrogenism [1, 21]. A retrospective study has been performed in patients with irregular cycles showing that treatment with DCI improves indexes of insulin resistance along with an increase of the percentage of women reporting regular menstrual cycles which was directly proportional to the increased duration of DCI treatment (24% and 51.6% at a mean of 6 and 15 months of treatment) [46]. One gr of DCI/die plus 400 mcg of folic acid daily* per os* for 6 months significantly improved insulin resistance as measured by HOMA Index (*p* = 0.001) and glycemia/insulin resistance index (IRI) ratio (*p* = 0.001). In the same study an improvement of systolic blood pressure (*p* = 0.001), Ferriman-Gallwey score (*p* = 0.001), LH (*p* = 0.001), LH/FSH ratio (*p* = 0.001), total testosterone (*p* = 0.001), free testosterone (*p* = 0.001), Δ-4-Androstenedione (*p* = 0.026), Prolactin (*p* = 0.010), and sex hormone binding globulin (*p* = 0.001) has been reported [22] (Table 1).

Based on the current evidence, it is widely accepted that both MYO-INS alone and DCI alone and their combination may have a beneficial effect on metabolic derangements associated to PCOS. Although there is no robust consensus regarding the ideal dosage of MYO-INS and/or DCI for PCOS treatment, a combination of both MYO-INS and DCI has been suggested by the promising results from studies with their combination [47]. The “International Consensus Conference on myo-inositol and D-chiro-inositol in Obstetrics and Gynecology” [48] suggests administering MYO-INS and DCI in a proposed “physiological ratio” of 40 : 1 based on the assumption that plasma ratio of MYO-INS/DCI in normal subjects is 40 : 1. However, the “International Consensus Conference on myo-inositol and D-chiro-inositol in Obstetrics and Gynecology” also suggests not giving high dose of DCI for the treatment of PCOS in order to avoid the negative effect of high dose of DCI on the ovary (the so-called DCI paradox).

## 3. Inositol and Other Insulin Resistance States

MYO-INS has been administered to postmenopausal women in order to investigate the effect on metabolic parameters. A prospective study assessed the effect of MYO-INS 2 g BID plus diet in postmenopausal women reporting a significant decrease of 75% of HOMA-IR index along with other metabolic markers (triglycerides, HDL cholesterol, and diastolic blood pressure) [23]. Patients were randomized to receive MYO-INS 2 g BID or placebo for 12 months. At the end of the study there was an improvement of all the metabolic parameters such as glucose, insulin, HOMA-IR (Homeostasis Model Assessment-Insulin Resistance), triglycerides, total and high density lipoprotein cholesterol, body mass index (BMI), waist circumference, and blood pressure [24]. Pregnant women with gestational diabetes were randomized to receive MYO-INS supplementation (4 g daily) plus folic acid (400 *μ*g daily) or folic acid only (400 *μ*g daily) as supplement to controlled diet for 8 weeks. Fasting glucose (*p* < 0.05), insulin (*p* < 0.05), and consequently HOMA-IR (*p* < 0.05) index significantly improved in both groups, although the improvement was greater in women treated with MYO-INS. Further, the treatment with MYO-INS was accompanied by an increase in adiponectin levels, while there was a decrease of adiponectin levels in the control group [25]. A two-year, prospective, randomized, open-label, placebo-controlled study was carried out on pregnant outpatients with a family history of type 2 diabetes who were randomized to receive 2 g MYO-INS plus 200 *μ*g folic acid or only 200 *μ*g folic acid twice a day from the end of the first trimester. MYO-INS supplementation significantly reduced the incidence of gestational diabetes (*p* = 0.04) and the delivery of macrosomia fetuses (*p* = 0.007) in pregnant women with a family history of type 2 diabetes [26] (Table 1).

Human evidence also has been confirmed by basic studies. The administration of sequoyitol, that is, the 5-O-methyl form of MYO-INS, (80 mg/kg per day) for 8 and 10 weeks, had antidiabetic effects in mice when administered chronically. In fact, both subcutaneous and oral administrations of sequoyitol improved glucose derangements and enhanced insulin signaling in liver of ob/ob insulin resistant mice [49]. The reduction of glucose levels after glucose load has been reported in healthy mice after both acute [50] and chronic administration of MYO-INS [51].

This effect was due to an improvement in peripheral insulin sensitivity that has been observed* in vivo* during an insulin tolerance test along with an enhanced GLUT-4 translocation to the plasma membrane in response to hyperglycemia at the skeletal muscle level [50].

## 4. Conclusion

The insulin-mimetic properties of dietary inositol are due to the production of inositol glycan secondary messengers containing either MYO-INS or DCI. Although randomized control trials using MYO-INS and/or DCI as supplement gave positive results in improving insulin resistance and reducing cardiovascular risk factors in women with PCOS and gestational diabetes mellitus or metabolic syndrome postmenopause, larger studies including both genders are needed in order to extend a possible application for a more generalized population at risk of developing or already presenting insulin resistance.

## Figures and Tables

**Table 1:** Clinical studies in which MYO or DCI supplementation has been evaluated for the treatment of disease associated to insulin resistance.

Authors, year	Inositol isomers	Duration of treatment	Experimental models
Genazzani et al., 2008 [17]	MYO 2 gr	12 weeks	PCOS
Genazzani et al., 2012 [18]	MYO 2 gr	8 weeks	PCOS
Gerli et al., 2007 [19]	MYO 4 gr	14 weeks	PCOS
Artini et al., 2013 [20]	MYO 2 gr	12 weeks	PCOS
Iuorno et al., 2002 [21]	DCI 600 mg	6 to 8 weeks	PCOS
Laganà et al., 2015 [22]	DCI 1 gr	6 months	PCOS
Giordano et al., 2011 [23]	MYO 2 gr BID	6 months	Postmenopausal women with metabolic syndrome
Santamaria et al., 2012 [24]	MYO 2 gr BID	12 months	Postmenopausal women with metabolic syndrome
Corrado et al., 2011 [25]	MYO 4 gr	8 weeks	Pregnant women with gestational diabetes
D'Anna et al., 2013 [26]	MYO 2 gr	6 months	Pregnant women with a family history of type 2 diabetes

*Note.* MYO: myo-inositol; DCI: D-chiroinositol.
